# High Levels of Prebiotic Resistant Starch in Diet Modulate Gene Expression and Metabolomic Profile in Pancreatic Cancer Xenograft Mice

**DOI:** 10.3390/nu11040709

**Published:** 2019-03-27

**Authors:** Concetta Panebianco, Annacandida Villani, Valerio Pazienza

**Affiliations:** Gastroenterology Unit, Fondazione IRCCS Casa Sollievo della Sofferenza, 71013 San Giovanni Rotondo, Italy; panebianco.c@gmail.com (C.P.); villaniannacandida@yahoo.it (A.V.)

**Keywords:** resistant starch, prebiotics, metabolomics, pancreatic cancer

## Abstract

Cancer initiation and protection mainly derives from a systemic metabolic environment regulated by dietary patterns. Less is known about the impact of nutritional interventions in people with a diagnosis of cancer. The aim of our study was to investigate the effect of a diet rich in resistant starch (RS) on cell pathways modulation and metabolomic phenotype in pancreatic cancer xenograft mice. RNA-Seq experiments on tumor tissue showed that 25 genes resulted in dysregulated pancreatic cancer in mice fed with an RS diet, as compared to those fed with control diet. Moreover, in these two different mice groups, six serum metabolites were deregulated as detected by LC–MS analysis. A bioinformatic prediction analysis showed the involvement of the differentially expressed genes on insulin receptor signaling, circadian rhythm signaling, and cancer drug resistance among the three top canonical pathways, whilst cell death and survival, gene expression, and neurological disease were among the three top disease and biological functions. These findings shed light on the genomic and metabolic phenotype, contributing to the knowledge of the mechanisms through which RS may act as a potential supportive approach for enhancing the efficacy of existing cancer treatments.

## 1. Introduction

Epidemiologic studies revealed associations between aspects of diet, nutrition, and physical activity with cancers, which are now the second most important cause of death globally, after cardiovascular disease, considering all of the non-communicable diseases (http://www.who.int/en/news-room/fact-sheets/detail/cancer). About one in six deaths is due to cancer worldwide, and it is estimated that from the 9.6 million deaths in 2018, there will be 23.6 million new cases of cancer each year by 2030. According to the World Health Organization (WHO) data, the major cancer risk factors are an unhealthy diet, high body mass index, tobacco use, alcohol use, and physical inactivity [1]. 

Like many cancers, pancreatic cancer incidence is also expected to rise in the near future. It is currently the third leading cause of cancer death worldwide [2], and it is expected to become the second by 2030 [3]. With a five-year survival rate of less than 5% for patients with a resectable disease [4], and an average survival time of six months for patients with advanced metastasized disease [5], pancreatic cancer is one of the most aggressive and fatal malignancies, with a tremendously poor prognosis. The reason for this extremely short survival time may be due to the very slow evolution of the disease, lasting up to 18 years from the time of the initiating mutations in pre-cancerous lesions to the metastatic disease [6]. 

As with many other cancers, environmental risks play a major role in pancreatic cancer. Only 5% to 10% of pancreatic cancer cases are hereditary, and the remaining 90% to 95% are due to environmental risk factors [7,8].

Recently, an international panel of recognized pancreatologists paid particular attention to the topic of nutritional support in the frame of pancreatic cancer for both short- and long-term outcomes [9], as diet, with its nutritional factors, powerfully modulates cells’ growth, homeostasis, and cancer promoting/preventing pathways. Moreover, diet is a modifiable target for the primary prevention and secondary management of pancreatic cancer. Among the nutrients, particular interest is addressed to glucose as a well-known fuel of cancer, as explained by the Warburg effect [10]. In a previously published study, we tested the effects of a diet enriched in RS on the growth of pancreatic cancer in xenografted mice. In this diet, corn starch, which is metabolized in the small intestine to release glucose, was replaced by RS, which instead reaches the large bowel undigested, so that glucose it contains is not released nor absorbed into the blood. We already demonstrated that, among the other beneficial effects on the gut microbiota composition, the tumor growth was significantly reduced in the mice fed with RS compared with the mice fed with a control diet [11]. In this study, we investigate the effect of this diet with a high content of RS on the gene expression and metabolomics profile, and its implication on cell pathways modulation in pancreatic cancer xenograft mice.

## 2. Materials and Methods

### 2.1. Animal Study

The animal study was carried out in an Association for Assessment and Accreditation of Laboratory Animal Care International (AAALAC) accredited experimental facility. The animal protocols were approved by the Institutional Animal Care and Use Committee, with approval number ANM14-002/468862. Twelve Nu/Nu nude female mice of five to six weeks of age were subcutaneously injected (right flank) with 5 × 10^6^ BxPC-3-luc cancer cells suspended in 0.1 mL of a Phosphate Buffered Saline (PBS)/matrigel mixture (1:1) per mouse.

When the tumor size reached an average volume of 100 mm^3^, the BxPC-3-luc tumor-bearing nude mice were randomly assigned into two groups (six mice/group), as follows: group 1 (standard diet) and group 2 (RS diet), as reported before [11]. The RS diet consisted of replacing the entire corn starch with type-2 resistant starch (Hi Maize 260^®^) [11]. The animals had free access to water. 

### 2.2. Preparation of RNA for RNAseq

The total RNA was extracted from the two control pancreatic cancer tissues and two pancreatic cancer tissues of the mice fed with a high RS diet, using a RNeasy mini kit (Qiagen, Milan, Italy), according to the manufacturer’s protocol. Then, 2 mg of purified RNA were used to prepare the indexed libraries, using a TruSeq Total Stranded RNA Sample Prep Kit (Illumina, Milan, Italy), according to the manufacturer’s instructions. After quantification on an Agilent 2100 Bioanalyzer (Agilent Technologies, Cernusco sul Naviglio, Italy), the libraries were pooled so that each index-tagged sample was present in equimolar amounts, with a final concentration of 2 nmol/L. The pooled samples were then subjected to cluster generation and were sequenced on an Illumina HiSeq 2500 System (Illumina, Milan, Italy) in a 2 x 100 paired-end format, at a final concentration of 8 pmol/L. The short reads were aligned against the hg19 genome assembly using STAR (version 2.6) with standard parameters, considering the genome features extracted from the UCSC RefSeq gtf file. The htseq-count tools were used to count the piled up reads. Using the edgeR R package (version 3.7), the counts were normalized (counts per million, CPM) and compared between the two contrasts (RS and control).

### 2.3 Ingenuity Pathway Analysis

The function “Core Analysis” included in the Ingenuity Pathway Analysis software (IPA; QIAGEN, http://www.ingenuity.com/) was used to perform an in silico analysis of the diseases, biological functions, and canonical pathways, in which the differential expression genes were involved. 

### 2.4. LC–MS Analysis of Serum Metabolomics

After precipitating the serum proteins using acetonitrile, the supernatant was diluted 1:1 in an acetonitrile/milliQ water mixture. The serum metabolites were analyzed using liquid chromatography/mass spectrometry by a slightly modified version of the acidic protocol, as described before [12]. A mixed pooled sample was created by taking a small aliquot from each sample for the quality control. A targeted approach was used to extract the response of the compounds included in a standard list of 97 compounds.

### 2.5. Statistical Analysis

A statistical analysis to identify the differentially expressed genes was performed using a multivariate regression model on the normalized data. A gene was considered differentially expressed if it showed a |fold-change| ≥1.5 and an adjusted *p*-value ≤0.05, as previously reported [13]. A functional enrichment analysis was performed by IPA (IPA, QIAGEN), using the following parameters:

- Reference set: ingenuity knowledge base (gene only);
- Relationship to include: direct and indirect;
- Filter: consider only the molecules and/or relationship where species = human, and confidence = experimentally observed.

Only the biological function and pathways showing a *p*-value ≤0.05 were considered.

For the metabolomic experiments, the results are expressed as mean ± standard deviation (SD). Comparisons were made using Student’s *t*-test. Differences were considered as significant when *p* < 0.05 (*) or *p* < 0.01 (**) or *p* < 0.001 (***).

## 3. Results

### 3.1. Differential Expression of Genes in PC Xenograft Mice under RS Diet

**Figure 1:**
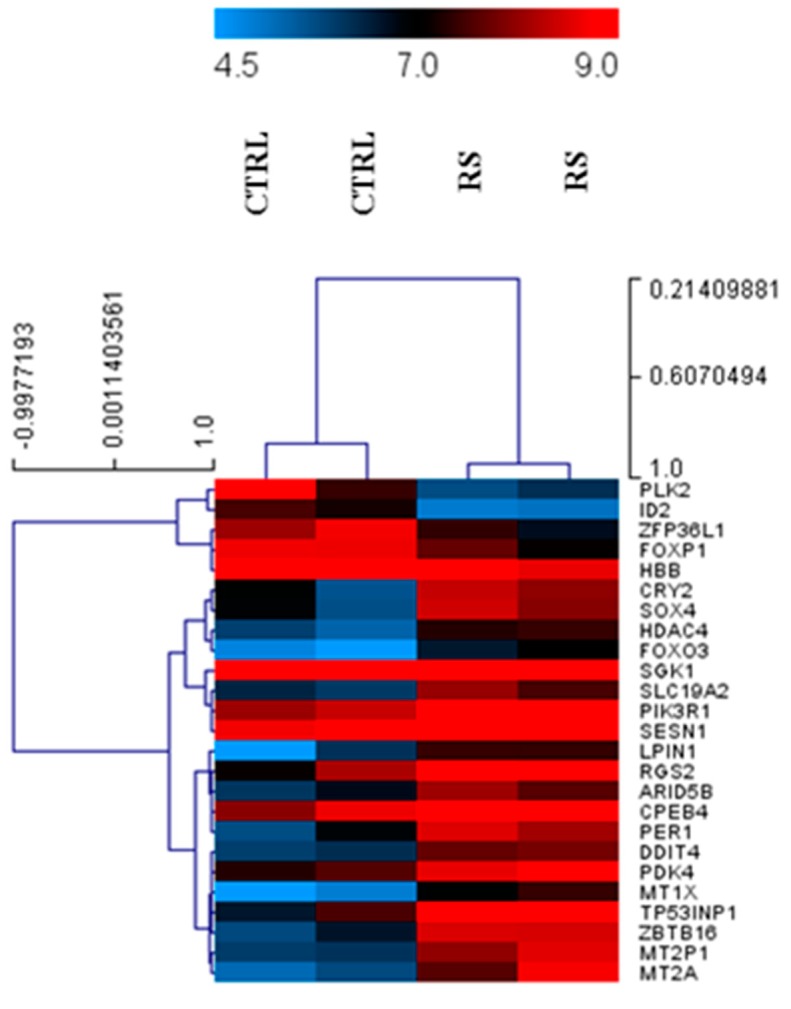
Heatmap of differentially expressed genes in pancreatic cancer tissue from the mice fed the control diet (CTRL) and the mice fed with the resistant starch (RS) diet.

As a first step, we analyzed the gene expression profiles in PC xenograft mice fed with the control diet and the RS diet. As shown in Figure 1, PLK2, ID2, ZFP36L1, FOXP1, and HBB resulted in being significantly down-regulated in the mice fed with an RS diet, compared with the control mice. On the other hand, PIK3R1, PDK4, ARID5B, CPEB4, RGS2, SGK1, LPIN1, HDAC4, SLC19A2, CRY2, SOX4, DDIT4, FOXO3, PER1, SESN1, TP53INP1, MT1X, MT2P1, ZBTB16, and MT2A were significantly up-regulated in the mice fed with the RS diet, with respect to the control mice.

### 3.2. Canonical Pathways and Disease and Biological Functions Analysis

**Figure 2:**
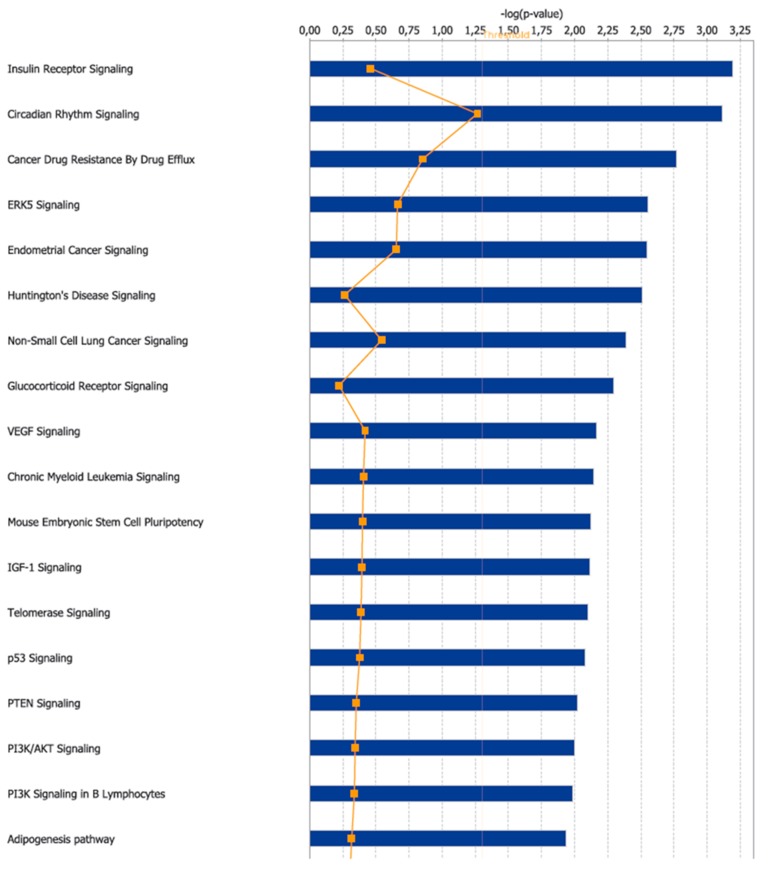
Top 18 canonical pathways enriched by the Ingenuity Pathway Analysis software (IPA) “Core Analysis” for the 25 genes differentially expressed between the CTRL and RS groups.

**Figure 3:**
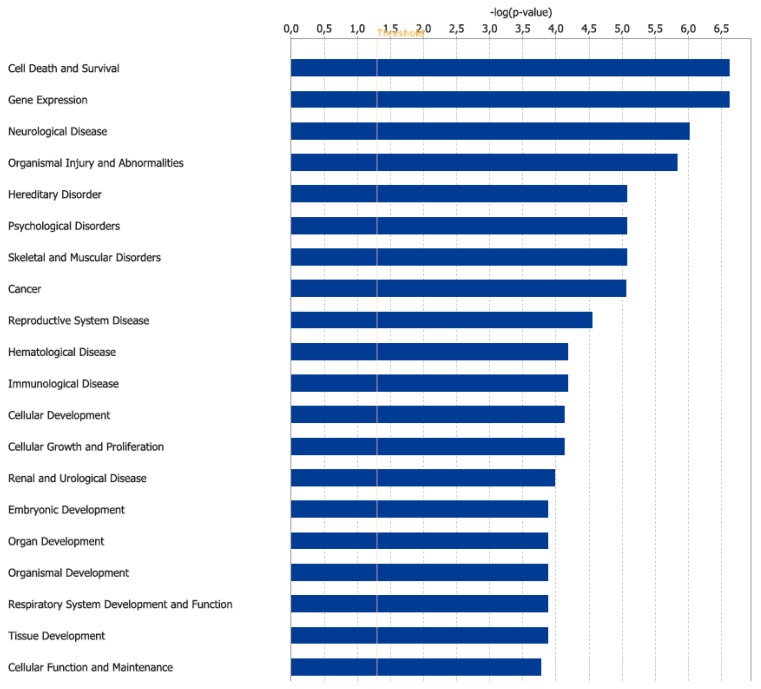
Top 20 diseases and biological functions enriched by the IPA “Core Analysis” for the 25 genes differentially expressed between the CTRL and RS groups.

**Figure 4:**
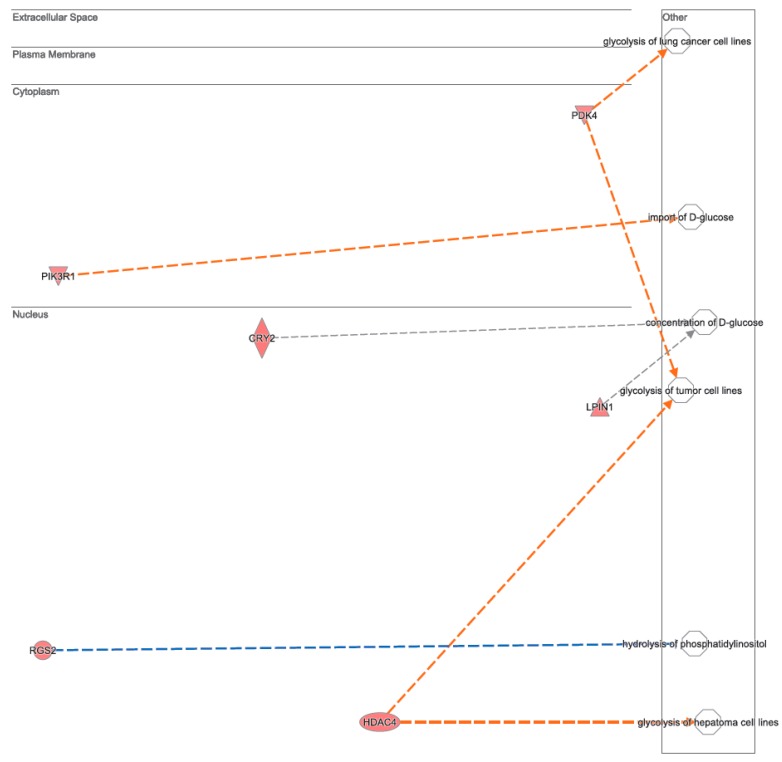
IPA network analysis of the genes differentially expressed between the CTRL and RS groups involved in carbohydrate metabolism. The genes shaded in red are up-regulated in RS compared with the CTRL group. The orange dotted arrows indicate activation, the blue dotted arrows indicate inhibition, and the grey dotted arrows indicate no predicted effect on the downstream biological processes.

**Figure 5:**
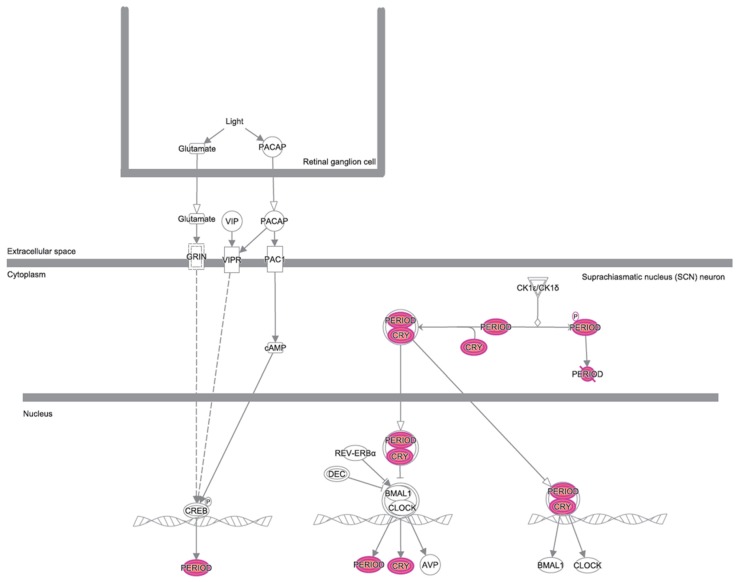
The circadian rhythm pathway identified as one of the significant pathways by the IPA. The genes of the up-regulated RS vs. CTRL are highlighted in pink.

A bioinformatic prediction related to the differential expression patterns placed insulin receptor signaling, circadian rhythm signaling, and cancer drug resistance among the three top canonical pathways (Figure 2), whilst, cell death and survival, gene expression, and neurological disease were placed among the three top disease and functions pathways (Figure 3). Figure 4 graphically represents the carbohydrate metabolism network involving some of the genes whose expression is changed in the mice under RS. Figure 5 graphically represents the pathway of the circadian rhythm that resulted in being affected by the RS diet.

### 3.3. Metabolomic Profile in PC Xenograft Mice under RS Diet

**Figure 6:**
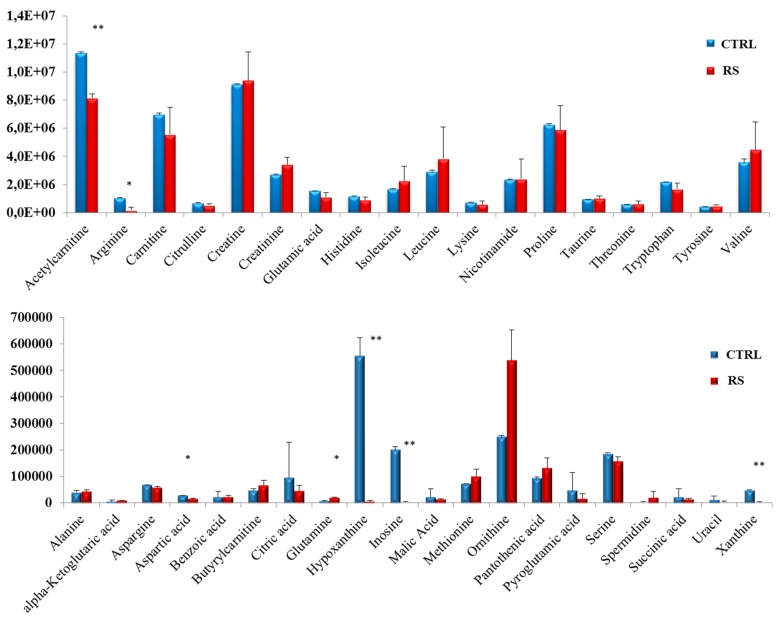
Bar chart representation of the 38 metabolites detected in the control and the RS-fed mice serum samples. Results are expressed as means ± standard deviation (SD). Differences were considered significant when *p* < 0.05 (*) or *p* < 0.01 (**).

We next evaluated the metabolomic profile of the serum samples collected from both the control mice and the mice fed with an RS diet. A total of 38 compounds were detected in the mice sera, namely: acetylcarnitine, arginine, carnitine, citrulline, creatine, creatinine, glutamic acid, histidine, isoleucine, leucine, lysine, nicotinamide, proline, taurine, threonine, tryptophan, tyrosine, valine, alanine, α-ketoglutaric acid, aspargine, aspartic acid, benzoic acid, butyrylcarnitine, citric acid, glutamine, hypoxanthine, inosine, malic acid, methionine, ornithine, pantothenic acid, pyroglutamic acid, serine, spermidine, succinic acid, uracil, and xanthine. Among these compounds, a significant decrease in acetylcarnitine, arginine, aspartic acid, hypoxanthine, inosine, and xantine was observed in the RS group, compared with the control group, while the level of glutamine results were increased (Figure 6).

## 4. Discussion

To date, it is well recognized that some nutritional exposures (alcohol and processed meat) are likely causal factors, but no singular factor protects against cancer (except for dietary fiber for colorectal cancer) [14]. However, avoiding excess adiposity and sedentary behaviors can impact on the features of cancer cells. Healthy cell replication and tissue integrity contributes to cancer protection. Nutritional state is a key factor involved in cancer development, progression, and outcome of the therapy [15]. Healthy dietary patterns are universally considered as those rich in fibers and plant foods (wholegrains, vegetables, legumes, and fruits) and fish, with a very modest consumption of meat, alcohol, and salt preserved foods. Among the dietary fibers, resistant starches are commonly considered to trigger health benefits, shaping the gut microbiota as a prebiotic, enhancing the production of short chain fatty acids (SCFA) [16,17], also impacting the immune system [18,19]. Our study aimed to identify the molecular mechanisms and cellular pathways affected by RS that could potentially benefit pancreatic cancer. As a first step, we performed a gene expression analysis on pancreatic cancer xenograft mice subjected to a high level of RS in their diet, compared to mice fed with a control diet. The resulting 25 genes deregulated by the RS diet were then analyzed with IPA. If the impact on the insulin receptor signaling was somehow to be expected, based on the reduced glucose levels made available by RS, it was interesting to note that this diet could also affect cell death and survival, cancer, mechanisms of anti-cancer drug resistance, and the circadian rhythm signaling, whose dysfunctions in tumors are well documented [20,21,22]. In detail, RS increased the expression of PER1 and CRY2, two of the clock genes activated by the formation of the CLOCK-BMAL1 heterodimer, and rhythmically controlling cellular processes [23]. Moreover, a three-fold down-regulation of the gene ID2 under RS was also observed. ID2 encodes a transcriptional repressor rhythmically interfering with the formation of the CLOCK-BMAL1 heterodimer [23]. It has been demonstrated that clock genes, including PER1 and CRY2, are down-regulated [24], whereas ID2 is over-expressed in pancreatic cancers [25,26]. Therefore, our results suggest that RS may counteract the deregulation of the peripheral circadian system occurring in pancreatic cancer. Actually, this is not the first report about nutrients regulating the circadian rhythm—glucose, amino acids, and alcohol have already been described as doing so, thus suggesting the existence of metabolic factors being able to integrate the nutrient signals with the biological clock [27]. 

Among the other genes deregulated by the RS diet, some genes involved in carbohydrate (and lipid) metabolism can be recognized. In detail, the up-regulation of the thiamine transporter SLC19A2 and of the PDK4 gene encoding a pyruvate dehydrogenase kinase, suggests that RS influences the oxidative decarboxylation reactions, as thiamine’s derivative thiamine pyrophosphate and PDK4 are a cofactor and an inhibitor of pyruvate dehydrogenase, respectively. The pyruvate dehydrogenase complex is regulated by nutritional status, and a reduced glucose availability is known to up-regulate PDKs expression, in particular PDK4, in order to inhibit glucose consumption [28]. PDK4 activation, in turn, promotes fatty acid oxidation to generate energy [29]. Also, the LPIN1 gene, which is induced by glucose deprivation [30], was up-regulated in the RS-fed mice. LPIN1 encodes a protein with a dual function in lipid metabolism—it inhibits fatty acid oxidation under normal glucose conditions, while it stimulates fatty acid oxidation in conditions of glucose depletion [30]. Furthermore, a number of genes whose expressions are influenced by the RS diet are regulators of tumor cell cycle and proliferation, such as PLK2 [31], FOXP1 [32], PIK3R1 [33], DDIT4 [34], SESN1 [35], and ZBTB16 [36], or regulators of apoptosis, such as SOX4 [37], FOXO3 [38], and TP53INP1 [39], suggesting that RS or dietary manipulation may act synergistically with anti-cancer therapy to improve the treatment of cancer patients.

Subsequently, we performed a metabolomic analysis on the serum of the mice on the control or RS diet. The most striking result was the drop in the level of the purine compounds inosine, hypoxanthine, and xanthine. The purine metabolism is strongly connected to cancer, and high levels of purine metabolites have been found in tumor cells. In particular, inosine and hypoxanthine are intermediates in the salvage pathway, which is the main source of purines within the cell [40]. Therefore, the remarkable decrease of these compounds observed in the blood of the mice under the RS diet may interfere with cancer cells’ proliferation. The significant increase in the glutamine levels observed under the RS-fed mice is noteworthy, given the prominent role of this amino-acid in supporting PC growth [41,42]. Like glucose, glutamine, whose oxidation produces glutamate and then α-ketoglutarate, provides intermediates for the Krebs’ cycle, thus contributing to fuel cell growth [43]. The glucose and glutamine metabolisms cooperate with each other [43,44], with glycolysis promoting the glutamine uptake and utilization [45]. In vitro experiments show that in the presence of low glucose levels, glutamine consumption decreases, and consequently, the glutamine levels in the medium increase [43]. Consistently, an in vivo study demonstrated that PC progression correlates with increased uptake and consumption, and reduced circulating levels of glutamine [46]. In light of these observations, we hypothesize that the lower glucose availability occurring in the RS-fed mice decreases the glutamine uptake and utilization by the cells, thus resulting in its accumulation in the serum. 

Overall, our results confirm that RS, along with other nutrients, has the potential to modulate gene expression and metabolism, potentially impacting the course of cancer disease. 

## 5. Conclusions

Promoting healthy ways of living through public education and information is in urgent need in order to adopt healthy behaviors to prevent or modify the disease’ outcome. Given their ability to modify many aspects of human biology (including gene expression, metabolism, microbiota, and immunity), the nutrients contained within the food that we consume represent one of the main factors influencing our well-being. Moreover, diet is an easily modifiable factor. The regular intake of foods with a high fiber content is considered a cornerstone of a healthy dietary pattern, and promises to have a potential benefit not only in preventing diseases, but also in ameliorating their course.
